# Ligand-dependent pharmacokinetic modulation via copper doping in ultrasmall gold nanoparticles

**DOI:** 10.1016/j.mtbio.2026.103009

**Published:** 2026-03-11

**Authors:** Jingqi Gong, Jiali Tian, Nengjie Wang, Di Huang, Yuanli Liu, Bing Tang

**Affiliations:** a College of Chemistry and Bioengineering, Guilin University of Technology, Guilin, 541006, People's Republic of China; b College of Materials Science and Engineering, Guilin University of Technology, Guilin, 541006, People's Republic of China

**Keywords:** Ultrasmall gold nanoparticles, Copper doping, Renal clearance, Tumor accumulation

## Abstract

Ultrasmall gold nanoparticles (AuNPs, <3 nm) exhibit great potential for precision nanomedicine. While ligand engineering and core doping have each been explored to enhance their performance, whether surface chemistry dictates the in vivo efficacy of alloyed nanoparticles remains unknown. To address this question, we used glutathione (GSH) and polyethylene glycol (PEG) as model stabilizing ligands, systematically comparing four corresponding nanoparticle groups: GSH-coated or PEGylated AuNPs, each with or without copper doping at comparable levels. Strikingly, copper doping induced negligible pharmacokinetic changes in PEGylated nanoparticles but significantly enhanced GSH-coated nanoparticles. GS-AuCuNPs exhibited significantly enhanced pharmacokinetics compared to undoped GS-AuNPs, with a 1.6-fold increase in the area under the plasma concentration-time curve. Tumor accumulation efficiency improved by 1.6-fold at 1 h and 1.5-fold at 12 h post-injection, accompanied by reduced renal excretion. This ligand-specific enhancement arises from a copper-induced interfacial reconfiguration, characterized by enhanced COO^−^–metal anchoring and increased exposure of –NH_2_ groups of GSH, which together increase the surface positive charge. These interfacial changes modulate nanoparticle–biological interactions, resulting in prolonged circulation and reduced renal excretion while preserving the intrinsic clearance pathway. Our findings reveal a core-ligand coupling principle whereby metal doping becomes pharmacokinetically effective only when paired with a chemically synergistic ligand shell.

## Highlights

• Copper doping shows a ligand-dependent pharmacokinetic effect, pronounced for glutathione-coated ultrasmall gold nanoparticles and minimal for PEGylated nanoparticles.
• In glutathione-coated ultrasmall gold nanoparticles, copper doping reduces renal excretion, prolongs blood exposure, and leads to enhanced tumor accumulation in vivo.
• These results reveal a core and ligand coupling principle for designing ultrasmall nanoparticles with tunable circulation and targeting.

## Graphical abstract

Copper doping exerts a ligand-dependent pharmacokinetic effect in ultrasmall nanoparticles. While negligible in PEGylated nanoparticles, it significantly prolongs blood circulation, enhances tumor targeting, and modulates renal clearance in glutathione-coated nanoparticles, elucidating a core–ligand synergy principle for rational nanomedicine design.

**Figure:**
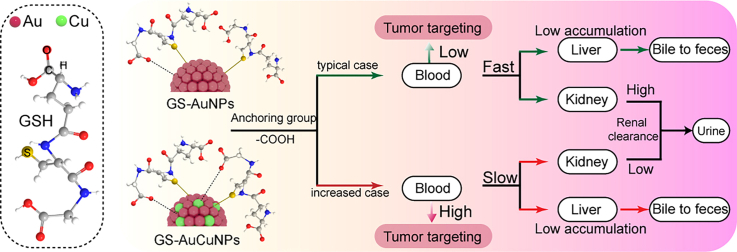

## 1 Introduction

Ultrasmall gold nanoparticles (AuNPs, <3 nm) demonstrate unique advantages in biomedical applications, particularly in drug delivery [1] and in vivo imaging [2], owing to their excellent biocompatibility, tunable surface chemistry, and efficient renal clearance capability [[3], [4], [5]]. Ultrasmall AuNPs with hydrodynamic diameter (HD) below the renal filtration threshold (KFT, ∼6 nm) undergo efficient glomerular clearance [6,7], facilitating rapid elimination from systemic circulation and substantially mitigating long-term bioaccumulation risks [8,9]. Surface ligand functionalization serves as a pivotal strategy to advance the application performance of ultrasmall AuNPs. Initially, glutathione (GSH), an endogenous zwitterionic tripeptide, is widely employed as a surface ligand for renal-clearable nanoparticles due to its biocompatibility and ability to enhance nanoparticle stability [[10], [11], [12]]. Subsequently, Poly(ethylene glycol) (PEG) functionalization, known as PEGylation, represents another well-established surface coating that provides steric stabilization and has been extensively used to modulate nanoparticle circulation [[13], [14], [15]]. Recent studies have shown that PEGylation of ∼3 nm citrate-stabilized AuNPs via thiolated PEG exchange can markedly prolong systemic retention [16,17]. While both GSH coating and PEGylation confer stability and favorable pharmacokinetics to ultrasmall AuNPs, the interplay between core composition, ligand chemistry, and in vivo fate remains incompletely understood, motivating strategies such as metal doping to further engineer nanoparticle behavior.

Metal doping offers a versatile means to enhance the functional performance of ultrasmall AuNPs [18]. Copper doping, in particular, enables precise tuning of electronic structures and introduces new functionalities [19,20], such as enhanced catalytic activity and near-infrared fluorescence [21]. Importantly, the effects of metal doping are inherently ligand-dependent, with nanoparticle behavior largely dictated by the surface ligand chemistry [22,23]. For instance, properties such as pH-responsive aggregation and photodynamic activity emerge only with specific coordinating ligands (e.g., mercaptosuccinic acid, MSA), whereas non-interacting coatings like PEG show minimal functional impact [24]. Recent work also suggests that MSA-coated AuNPs can exhibit reduced accumulation in MPS organs upon metal doping, highlighting subtle ligand–core interactions [25]. Nevertheless, most studies to date have focused on therapeutic endpoints or intrinsic material properties, and the ligand-dependent influence of copper doping on the pharmacokinetics of ultrasmall AuNPs remains largely unexplored. Understanding this doping–ligand synergy is critical for the rational design of next-generation nanomedicines that combine prolonged circulation, precise targeting, and improved safety.

Here, we directly address this unknown by constructing a precisely controlled model system that decouples the variables of core composition and surface chemistry. By employing both multidentate GSH and flexible PEG as representative yet chemically distinct ligands, we specifically test the hypothesis that the functional outcome of core doping is contingent on complementary chemical interactions at the interface. Four well-defined nanoparticle groups were synthesized: GSH-coated AuNPs (GS-AuNPs), GSH-coated copper-doped AuNPs (GS-AuCuNPs), PEGylated AuNPs (PEG-AuNPs), and PEGylated copper-doped AuNPs (PEG-AuCuNPs). Through a comparative analysis of their in vivo pharmacokinetics, we aim to determine whether the biological impact of copper doping is ligand-specific, particularly in terms of its ability to prolong circulation, maintain efficient clearance, and enhance tumor accumulation. This approach moves beyond the empirical use of stealth coatings and demonstrates that effective pharmacokinetic programming requires the rational pairing of a doped core with a chemically compatible ligand shell to form a synergistic unit.

## 2 Experiment section

### 2.1 Materials and reagents

The reduced glutathione (GSH) and poly (ethylene glycol) methyl ether thiol (PEG-SH) were purchased from Sigma-Aldrich; Gold chloride trihydrate (99%), copper chloride dihydrate (99.9%) and sodium borohydride were obtained from Meryer (Shanghai) Chemical Technology Co., Ltd. All reagents were used as received unless otherwise stated. Ultrapure water (PALL, USA, 18.2 MΩ cm^−1^) was used for the experiments. Before use, all glassware and Teflon-coated stir bars were washed with aqua regia (HCl/HNO_3_ = 3:1, *v*/*v*) and rinsed thoroughly with ultrapure water. The obtained nanoparticles were purified by ultrafiltration using 10 kDa MWCO filters (Millipore, USA).

### 2.2 Apparatus

UV–vis absorption spectra were recorded on a Shimadzu UV-2600 spectrophotometer (Shimadzu, Japan). Hydrodynamic diameters (HDs) were measured using a Zetasizer Nano ZS (Malvern). High-resolution transmission electron microscopy (HRTEM) images were obtained with a FEI Talos F200 × microscope operated at an accelerating voltage of 200 kV. For X-ray photoelectron spectroscopy (XPS) measurements, sample solutions were dried on aluminum foils and measured on an ESCALAB 250Xi instrument (Thermo Scientific, USA) with Al Kα X-ray radiation (1486.6 eV) as excitation source. All the binding energies were calibrated by C 1s as reference energy (284.8 eV). The XPS spectra were fitted with mixed Gaussian–Lorentzian functions using Thermo Avantage software (version 6.90). The Au content in the metal nanoparticles and biological samples was quantified by inductively coupled plasma mass spectrometry (ICP-MS) using a Thermo Scientific iCAP RQ instrument (USA).

### 2.3 Preparation of GS-AuNPs and GS-AuCuNPs

GS-AuNPs and GS-AuCuNPs were synthesized following a previously reported size-focusing strategy using NaBH_4_ as the reducing agent [26]. In a typical synthesis, an aqueous solution of the metal precursor, HAuCl_4_ (325 μL, 20 mM) or a mixed aqueous solution of HAuCl_4_ (286 μL, 20 mM) and CuCl_2_ (39 μL, 20 mM), was added to 4.415 mL of deionized water under vigorous stirring. Subsequently, an aqueous solution of glutathione (GSH, 260 μL, 0.1 M) was introduced. The reaction mixture turned bright yellow within seconds and then rapidly became colorless and transparent. Freshly prepared NaBH_4_ aqueous solution (0.5 mg/mL, 5 mL) was then added rapidly, upon which the solution immediately turned brown, indicating the formation of polydisperse GSH-stabilized AuNPs or AuCuNPs. The resulting dispersion was aged at room temperature for 5 h to afford size-focused GS-AuNPs and GS-AuCuNPs.

### 2.4 Preparation of PEG-AuNPs and PEG-AuCuNPs

PEG-AuNPs and PEG-AuCuNPs were synthesized following the same procedure as for GS-AuNPs and GS-AuCuNPs, except that GSH was replaced by PEG-SH.

### 2.5 Biodistribution and pharmacokinetics

The mice were purchased from the Hunan Sileike Jingda Laboratory Animal Co., Ltd. (Hunan, China), and housed in the 924th Hospital of the Chinese People's Liberation Army Joint Logistic Support Force in a 12 h light-dark cycle, with constant room temperature (23 ± 1 °C) and relative humidity (50 ± 5%). Healthy female Balb/c mice (5 weeks old) were used for pharmacokinetic studies, while female Balb/c nude mice (5 weeks old) bearing MDA-MB-231 xenografts were employed to assess tumor accumulation, as an immunodeficient host is required for stable growth of human xenografts. All the mice had free access to water and standard laboratory food. All experiments involving animals were conducted with approval from the Ethics Committee of Laboratory Animal of Institute of Animal Health, 924th Hospital of the Chinese People's Liberation Army Joint Logistic Support Force.

After intravenous (i.v.) injection of 200 μL AuNPs or AuCuNPs (10 mg/kg), the blood samples of normal mice (n = 3) were collected from the eye socket at 2, 3.5, 5, 10, 30 min, 1, 3, 5, 8, 12, 24, 48, and 72 h post-injection (p.i.), respectively. The blood samples were then weighed and digested with fresh aqua regia (HCl/HNO_3_ = 3:1, *v*/*v*) until the mixed solution evaporated completely. Finally, all samples were redissolved in aqua regia solution (3%, *v*/*v*) for ICP-MS quantification of Au concentration.

The 200 μL AuNPs or AuCuNPs (10 mg/kg) were i.v. injected into the subcutaneous MDA-MB-231 tumor-bearing mice. The mice were sacrificed at 1 and 12 h p.i., respectively, and the tumor together with organs were collected. The Au concentrations in the samples were measured following the same procedure as described above.

### 2.6 Tumor models

The female Balb/c mice (n = 3) were housed under standard environmental conditions (23 ± 1 °C, 50 ± 5% humidity and a 12/12 h light/dark cycle) with free access to water and standard laboratory food. The Balb/c nude mice were subcutaneously inoculated with MDA-MB-231 cells suspended in DMEM (2 × 10^6^ cells per mouse) to prepare subcutaneous MDA-MB-231 tumor models. This tumor model was chosen for its well-characterized vascular permeability, enabling nanoparticle accumulation via the enhanced permeability and retention (EPR) effect [27].

### 2.7 Statistical analyses

The data were shown as mean ± s.d. Statistical significance was determined using two-tailed unpaired Student's t-tests. ns, not significant; ∗*p* < 0.05; ∗∗*p* < 0.01; ∗∗∗*p* < 0.001; ∗∗∗∗*p* < 0.0001.

## 3 Results and discussion

### 3.1 Characterization of the ultrasmall AuNPs and AuCuNPs

**Fig. 1:**
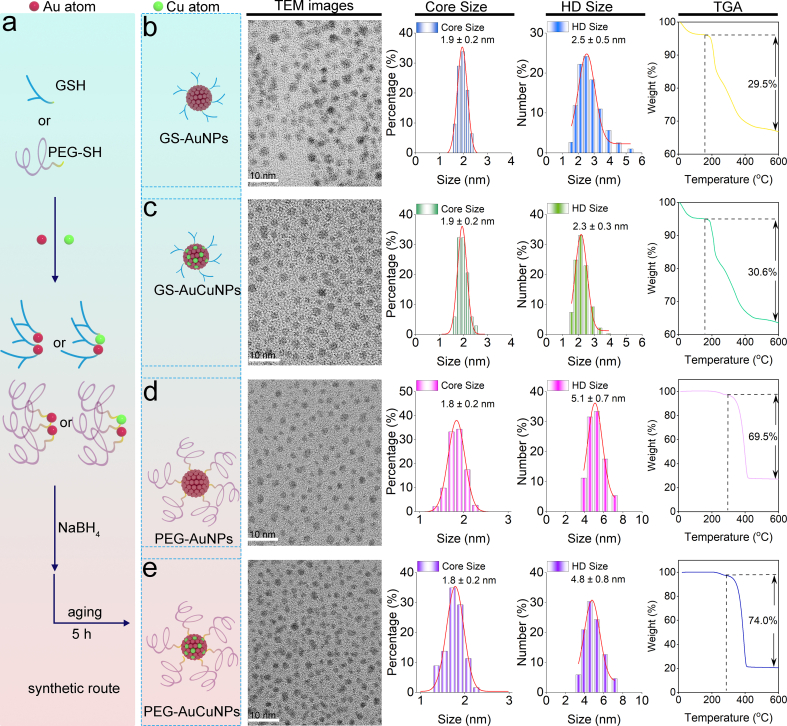
Characterization of GSH-coated and PEGylated ultrasmall AuNPs and AuCuNPs. (a) Scheme of the ultrasmall nanoparticle synthesis. Typical TEM images, Core size measured by TEM, HDs and TGA results for GS-AuNPs (b), GS-AuCuNPs (c), PEG-AuNPs (d) and PEG-AuCuNPs (e).

A series of ultrasmall nanoparticles were synthesized to directly assess the ligand-dependence of copper doping effects on the pharmacokinetics of ultrasmall AuNPs. Ultrasmall AuNPs and AuCuNPs were synthesized through a one-step aqueous reduction method [26]. The zwitterionic GS-AuNPs and PEG-AuNPs were synthesized by reducing HAuCl_4_ with NaBH_4_ in the presence of GSH and thiolated PEG (PEG-SH, MW 1 kDa), respectively. PEG (1 kDa) was selected based on literature demonstrating its stabilization of ultrasmall AuNPs [13]. For the AuCuNPs, a copper salt (CuCl_2_) was introduced during the co-reduction. Following the described protocol, GS-AuNPs, GS-AuCuNPs, PEG-AuNPs, and PEG-AuCuNPs were prepared (Fig. 1a). The core sizes of the ultrasmall nanoparticles were characterized by transmission electron microscopy (TEM). TEM confirmed the ultrasmall AuNPs and AuCuNPs with different surface coverages but identical core sizes (∼2.0 nm) (Fig. 1b–e). Quantitative analysis of high-resolution TEM micrographs yielded size statistics of 1.9 ± 0.2 nm (GS-AuNPs and GS-AuCuNPs) and 1.8 ± 0.2 nm (PEG-AuNPs and PEG-AuCuNPs). This negligible size variation eliminates size as a confounding variable, enabling unambiguous attribution of ligand-dependent biological behaviors of copper-doped nanoparticles to surface coating and copper incorporation. Furthermore, the hydrodynamic properties of the nanoparticles were analyzed by dynamic light scattering (DLS). HD demonstrated a clear segregation based on surface ligand type. PEGylated nanoparticles displayed larger HD (∼5.5 nm) of PEG-AuNPs (5.1 ± 0.7 nm) and PEG-AuCuNPs (4.8 ± 0.8 nm), consistent with the formation of a hydrated and extended polymeric corona as previous reports [13]. In contrast, nanoparticles coated with the compact GSH exhibited smaller HD for GS-AuNPs (2.5 ± 0.5 nm) and GS-AuCuNPs (2.3 ± 0.3 nm), respectively. Such a marked increase in HD after PEGylation, in comparison with zwitterionic GS coating, aligns well with established literature [13]. Critically, within each ligand category, the presence of copper doping did not lead to significant alterations in HD, thus preserving the intrinsic size-dependent properties of each ligand type and ensuring that subsequent biological differences arise from ligand-specific interfacial synergy rather than altered physical dimensions. TGA analysis revealed minor differences in surface ligand densities between doped and undoped nanoparticles within each ligand modification, with approximately 5.28 and 5.34 ligands/nm^2^ for GS-AuNPs and GS-AuCuNPs, respectively, and 8.52 and 10.02 ligands/nm^2^ for PEG-AuNPs and PEG-AuCuNPs (Table S1). With GSH and PEG modification, all nanoparticles displayed colloidal stability under physiological conditions (Figs. S1 and S2).

**Fig. 2:**
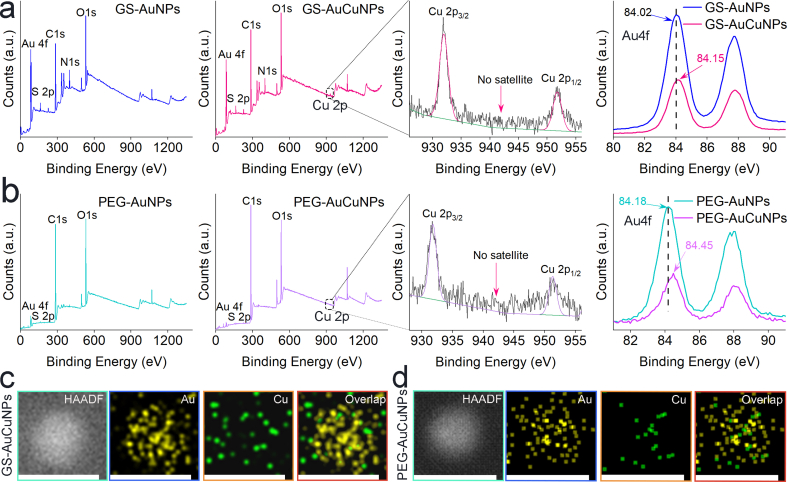
Compositional analysis of PEGylated and GSH-coated ultrasmall AuNPs and AuCuNPs. (a, b) XPS spectra of GSH-coated (a) and PEGylated (b) nanoparticles, including survey scans (left), high-resolution Cu 2p (right-middle), and Au 4f (right) spectra. (c, d) HAADF-STEM image and elemental EDS mapping of a single GS-AuCuNPs (c) and PEG-AuCuNPs (d), verifying formation of the alloy nanoparticles (Au: yellow; Cu: green).

X-ray photoelectron spectroscopy (XPS) was employed to probe the surface chemistry and verify successful copper doping. Survey spectra confirmed the presence of all expected elements, while an exclusive Cu signal in AuCuNPs verifies successful doping (Fig. 2a and b). High-resolution analysis revealed key changes consistent with alloy formation and specific interfacial bonding. The Cu 2p_3/2_ peak and the Cu 2p_1/2_ peak observed in both GS-AuCuNPs (931.96 eV,951.76 eV) and PEG-AuCuNPs (931.78 eV,951.30 eV), with the absence of satellite features, indicating the presence of Cu(0)/Cu(I) states [28] (Fig. 2a and Fig. S3). Furthermore, incorporation of Cu into the AuNPs induced a positive shift in the Au 4f binding energy by 0.13 eV for GS-AuCuNPs and 0.27 eV for PEG-AuCuNPs. Although the observed Au 4f shift is consistent with metallophilic interactions between Au(I) and Cu(I) [29], additional electronic contributions arising from alloy formation may also be involved [30]. In addition, scanning transmission electron microscopy (STEM) coupled with EDS mapping confirmed that both the GS-AuCuNPs (Fig. 2c) and PEG-AuCuNPs (Fig. 2d) contained both Au and Cu elements. Furthermore, the Cu/Au atomic ratios were quantitatively determined by inductively coupled plasma mass spectrometry (ICP-MS), yielding values of 0.21 for GS-AuCuNPs and 0.24 for PEG-AuCuNPs. These results collectively confirm successful and uniform copper doping within the alloy nanoparticles.

**Fig. 3:**
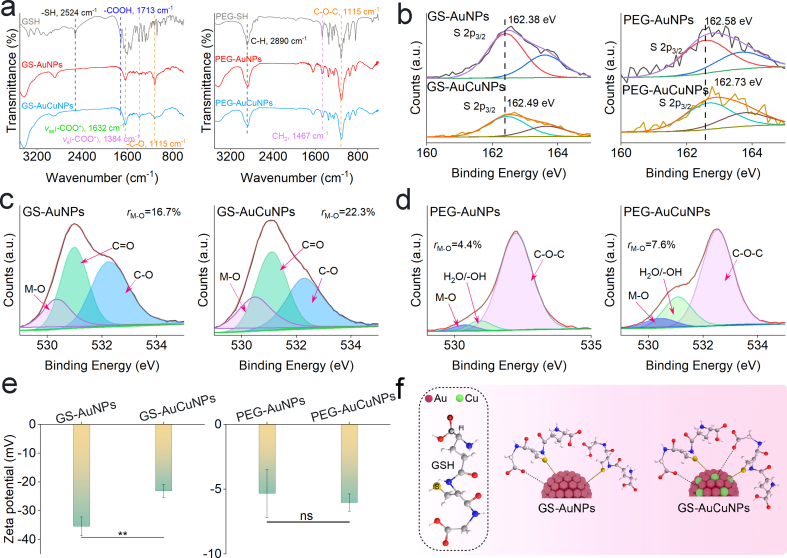
Enhanced ligand-metal interaction of ultrasmall AuCuNPs. (a) FT-IR spectra of GSH-coated and PEGylated nanoparticles. (b) High-resolution XPS spectra of S 2p for nanoparticles (insets: corresponding peak fitting results with marked binding energy shifts). (c, d) High-resolution XPS spectra of O 1s for GSH-coated (c) and PEGylated (d) nanoparticles. (e) Zeta potential measurements of GSH-coated and PEGylated nanoparticles at pH 7.4. (f) Schematic illustration of the surface coating structure for GS-AuNPs and GS-AuCuNPs. Data are presented as mean ± SD (n = 3). Statistical significance was determined using a two-tailed unpaired Student's t-test. ns, not significant; ∗*p* < 0.05; ∗∗*p* < 0.01; ∗∗∗*p* < 0.001; ∗∗∗∗*p* < 0.0001.

To elucidate whether copper doping modulates the ligand-core interface, we performed FTIR and XPS analyses on both GSH-coated and PEGylated nanoparticles with or without copper incorporation. FTIR spectroscopy revealed ligand-specific changes upon copper doping (Fig. 3a). For pristine GSH, characteristic bands at ∼2524 cm^−1^ and ∼1713 cm^−1^ correspond to the stretching vibrations of free -SH and -COOH groups, respectively [31]. After binding to AuNPs, the disappearance of the -SH band indicates Au-S bond formation, while the loss of the free -COOH groups and the emergence of peaks at ∼1632 cm^−1^ (ν_as_(COO^−^)) and ∼1384 cm^−1^ (ν_s_(COO^−^)) suggest COO^−^ coordination on the nanoparticle surface [32,33]. Compared with GS-AuNPs, GS-AuCuNPs exhibit increased intensities of the ν_as_ and ν_s_ for COO^−^ bands together with a reduced C-O stretching band (∼1115 cm^−1^) [34]. These differences indicate a redistribution of ligand upon copper incorporation, suggesting stronger interactions between the metal and -COO^-^ groups in GS-AuCuNPs. In contrast, PEGylated nanoparticles showed no significant spectral differences beyond the disappearance of the -SH band.

XPS spectra provided detailed insights into the ligand-metal interface chemistry. As shown in Fig. 3b, the S 2p_3/2_ main peak position of GS-AuCuNPs shifted to a higher binding energy (162.49 eV) compared to that of GS-AuNPs (162.38 eV), consistent with the incorporation of copper into the nanoparticle surface and the likely formation of Cu-S bonds [35]. A similar shift was observed in the PEGylated nanoparticles, where the S 2p_3/2_ peak shifted from 162.58 eV (PEG-AuNPs) to 162.73 eV (PEG-AuCuNPs). These systematic shifts in sulfur binding energy confirm the successful incorporation of copper into the nanoparticle surface. Quantification of the O 1s spectra further elucidated the interfacial reorganization upon copper doping (Fig. 3c and d). High-resolution O 1s XPS spectra of all samples revealed the presence of C-O, O=C, and metal–oxygen (M − O, M = Au, Cu) components [[36], [37], [38]]. Deconvolution revealed a significant increase in the M − O component area upon doping. The relative area of the M − O component increased from 16.7% in GS-AuNPs to 22.3% in GS-AuCuNPs. A similar trend was observed in the PEGylated nanoparticles, where the M − O contribution increased from 4.4% (PEG-AuNPs) to 7.6% (PEG-AuCuNPs). This increase in the M − O component indicates enhanced COO^−^ groups in metal coordination upon copper incorporation, confirming that copper doping actively participates in interfacial bonding and modulates the ligand configuration.

Zeta potential measurements further confirm this interfacial modulation. According to GSH containing anchoring groups of -COOH and -NH_2_, GS-AuCuNPs displayed a more positive surface potential (−23.1 ± 2.3 mV) than GS-AuNPs (−35.4 ± 3.3 mV), consistent with enhanced COO^−^–metal anchoring and increased exposure of –NH_2_ groups (Fig. 3e) [39]. However, PEG-AuCuNPs exhibited negligible potential changes relative to PEG-AuNPs, indicating that the surface charge modulation is specific to the GSH modification. In addition, the increased COO^−^–metal anchoring resulted in different performances in interaction with serum proteins (Fig. S4). Together with the FTIR and XPS results discussed above, these observations demonstrate that copper doping not only incorporates into the core but also actively participates in surface coordination, thereby altering the ligand configuration and surface charge (Fig. 3f). Such a reconfiguration is expected to modify the nanoparticle's biological interfacial properties [39,40], which motivated our subsequent investigation into its pharmacokinetic impact.

### 3.2 Pharmacokinetics and metabolism of the AuNPs and AuCuNPs

**Fig. 4:**
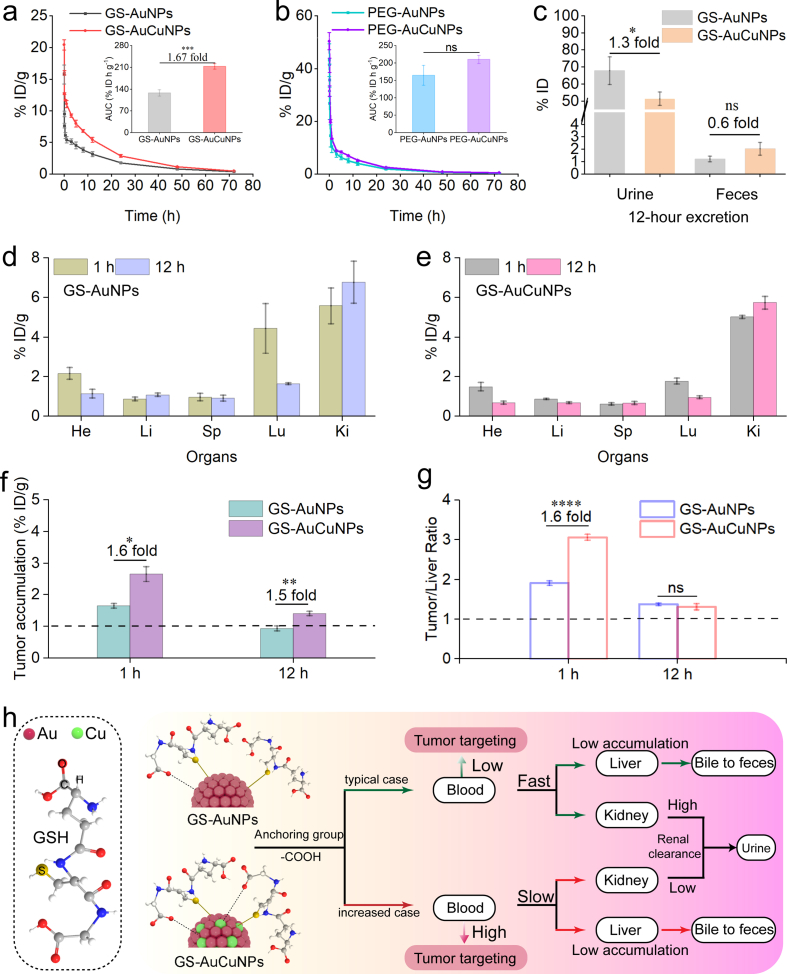
In vivo pharmacokinetics, biodistribution, and tumor-targeting behaviors of ultrasmall AuNPs and AuCuNPs. (a, b) Pharmacokinetics and corresponding AUC values (insets) for the GSH-coated (a) and PEGylated (b) ultrasmall AuNPs and AuCuNPs. (c) Renal and hepatic clearance of GS-AuCuNPs and GS-AuNPs at 12 h p.i. (d, e) Biodistributions in mice bearing MDA-MB-231 tumors of the GS-AuCuNPs (d) and GS-AuNPs (e) at 1 and 12 h p.i. (f) Corresponding tumor accumulation of both nanoparticles at the indicated time points. (g) Time-dependent tumor to liver ratios of GS-AuCuNPs and GS-AuNPs over 12 h p.i. (h) Schematic diagram summarizing the distinct in vivo behaviors of GS-AuCuNPs and GS-AuNPs. Data are presented as mean ± SD (n = 3). Statistical significance was determined using a two-tailed unpaired Student's t-test. ns, not significant; ∗*p* < 0.05; ∗∗*p* < 0.01; ∗∗∗*p* < 0.001; ∗∗∗∗*p* < 0.0001.

The prolonged circulation of PEGylated and GSH-coated ultrasmall AuNPs in the bloodstream facilitates their preferential accumulation in tumor tissues via the EPR effect, a critical determinant for effective tumor accumulation [13,41]. While two-compartment PK behavior was observed for all the AuNPs, the distribution (t_1/2α_) and elimination (t_1/2β_) half-lives were further analyzed (Table S2). Consistent with previous literature [13], PEG-AuNPs demonstrated substantially enhanced systemic exposure compared to that of GS-AuNPs. This was evident in a significantly larger area under the curve (AUC) for PEG-AuNPs (164.5 ± 28.5 %ID·h·g^−1^) relative to that of GS-AuNPs (127.8 ± 10.3 %ID·h·g^−1^), in a markedly prolonged t_1/2α_ in PEG-AuNPs (12.6 ± 3.6 min) versus that in GS-AuNPs (3.4 ± 0.35 min), and in a slight decrease in the t_1/2β_ (Fig. 4a and b).

Further analysis revealed that the pharmacokinetic modulation induced by copper doping is ligand-dependent. Copper doping exerted a profound and positive effect within the GSH-coated nanoparticles. GS-AuCuNPs demonstrated a significantly enhanced circulation profile relative to GS-AuNPs (Fig. 4a and Fig. S5). This was most evident in a marked increase in systemic exposure, with the AUC of GS-AuCuNPs reaching 212.9 ± 8.9 %ID·h·g^−1^, representing an approximately 1.67-fold increase over that of GS-AuNPs. Moreover, the t_1/2β_ was extended from 3.8 ± 0.56 h to 5.7 ± 0.43 h, which is within the time window commonly associated with enhanced passive tumor accumulation in EPR-reliant systems [41], thereby positioning GS-AuCuNPs as a promising candidate for enhanced tumor accumulation via passive targeting. Importantly, this enhancement cannot be attributed merely to the presence of copper ions. Control experiments (Fig. S6 and Table S2) with GS-AuNPs incubated with free copper ions (GS-AuNPs + Cu^2+^) showed a significant decrease in AUC, to 73.9 ± 10.7 %ID·h·g^−1^. This indicates that the improved pharmacokinetics require copper to be incorporated into the nanoparticle alloy during synthesis, rather than merely chelated by the surface ligand. In stark contrast, the PEG-AuCuNPs did not exhibit a statistically significant alteration in their pharmacokinetic profile relative to the PEG-AuNPs, with no meaningful differences observed in AUC, t_1/2α_, or t_1/2β_ (Fig. 4b and Table S2). The ligand-specific pharmacokinetic outcome demonstrates that while copper doping effectively enhances the pharmacokinetics of zwitterionic ligand-coated nanoparticles (e.g., GSH), it exhibits no significant effect on PEG-coated counterparts. This highlights the deterministic role of interfacial chemistry in governing the in vivo fate of alloyed nanoparticles.

The impact of copper doping with GS-AuNPs was further elucidated by analyzing the primary metabolic clearance pathways. Notably, urinary excretion of GS-AuCuNPs began to diverge from that of GS-AuNPs as early as 6 h p.i., with the difference becoming more pronounced by 12 h, where cumulative excretion decreased from 67.7 ± 8.1 %ID for GS-AuNPs to 51.3 ± 3.9 %ID for GS-AuCuNPs (Fig. 4c and Fig. S7). This distinct reduction in renal excretion corresponds directly to and mechanistically explains the observed pharmacokinetic enhancement, particularly the elevated AUC of GS-AuCuNPs relative to GS-AuNPs (Fig. 4a). Both ultrasmall nanoparticles exhibited a clearance profile dominated by high renal excretion and low hepatic clearance. Cumulative urinary excretion reached 82.5 ± 7.5% ID for GS-AuNPs and 69.9 ± 4.7% ID for GS-AuCuNPs by 168 h p.i., with consistently low fecal excretion throughout the study period (<10% ID, Fig. S8a). As the primary clearance organ, kidney sections showed no histological abnormalities, confirming the renal biosafety of all nanoparticle formulations (Fig. S9). Furthermore, they demonstrated high clearance efficiency, with minimal residual Au accumulation in major organs (heart, liver, spleen, lung, and kidneys) within 7 days p.i. (Fig. S8b), indicating favorable biosafety profiles.

### 3.3 Biodistribution and tumor target efficiency of the GS-AuNPs and GS-AuCuNPs

The prolonged circulation and attenuated renal excretion of GS-AuCuNPs prompted tumor-targeting evaluation at 1 h and 12 h [41], corresponding to the circulation and distribution phases; at both time points, minimal accumulation was observed in most major organs (Fig. 4d and e). Au content in tumor tissue gradually decreased from 1 to 12 h p.i. for both of them, consistent with systemic clearance, yet GS-AuCuNPs maintained significantly higher tumor accumulation than GS-AuNPs at 1 to 12 h p.i. (Fig. 4f). Specifically, GS-AuCuNPs exhibited approximately 1.6- and 1.5-fold higher tumor accumulation than GS-AuNPs at the respective time points. Additionally, we investigated the tumor accumulation specificity of GS-AuCuNPs and GS-AuNPs. GS-AuNPs show much higher targeting specificity at both 1 h and 12 h p.i. as literature reports [41], Notably, GS-AuCuNPs (3.1 ± 0.1) showed a 1.6-fold higher tumor-to-liver ratio than GS-AuNPs (1.9 ± 0.1) at 1 h p.i., further reflecting its enhanced targeting specificity relative to the major clearance organ (Fig. 4g). Importantly, despite a decrease in absolute accumulation over time, the tumor-to-liver ratio for both GS-AuCuNPs and GS-AuNPs remained above 1 at 12 h p.i., underscoring their sustained tumor-targeting capability and favorable selectivity over hepatic retention. Silver staining indicated the presence of both nanoparticles within tumor tissue (Fig. S10), and such ultrasmall nanoparticles may enter tumor cells via passive and/or active mechanisms as previously reported [42]. Furthermore, the copper-doping–dependent modulation of tumor accumulation and specificity was observed at more than one alloy composition, suggesting that the ligand-dependent targeting behavior is composition-sensitive rather than incidental (Fig. S11). These results establish that copper doping transforms the GSH-coated nanoparticle's biological trajectory, shifting distribution from fast renal excretion to high tumor accumulation while maintaining temporal clearance patterns (Fig. 4h). This ligand-dependent enhancement establishes a more favorable targeting profile with altering the fundamental clearance kinetics.

## 4 Conclusion

In summary, we demonstrate that the pharmacokinetic efficacy of core copper doping is ligand-dependent. In GSH-coated nanoparticles, copper incorporation promotes Cu-S and M−O coordination, induces interfacial reconfiguration with enhanced COO^−^-metal anchoring and increased -NH_2_ group exposure, and thereby modulates surface charge distribution to improve circulation and tumor accumulation. In contrast, copper doping does not induce significant surface potential reorganization of PEGylated nanoparticles, resulting in negligible pharmacokinetic alteration. These findings suggest that effective pharmacokinetic modulation by core doping requires functional coupling between alloy cores and chemically responsive ligand shells. In the context of established paradigms where ligand chemistry, surface charge, and dynamic protein corona formation govern nanoparticle fate in vivo, our results indicate that alloy composition can act as an additional regulatory factor by subtly reconfiguring the ligand–core interface in a ligand-dependent manner. While this study primarily examined tumor accumulation of GSH-coated nanoparticles, future work will evaluate all formulations across diverse tumor models and varying copper doping ratios to validate the generality and doping-level dependence of this ligand-mediated effect.

## CRediT authorship contribution statement

**Jingqi Gong:** Data curation, Investigation, Methodology. **Jiali Tian:** Data curation, Investigation, Methodology. **Nengjie Wang:** Investigation, Software. **Di Huang:** Formal analysis, Funding acquisition, Writing – original draft. **Yuanli Liu:** Resources, Supervision, Writing – review & editing. **Bing Tang:** Conceptualization, Formal analysis, Funding acquisition, Methodology, Project administration, Visualization, Writing – review & editing.

## Declaration of competing interest

The authors declare that they have no known competing financial interests or personal relationships that could have appeared to influence the work reported in this paper.

## Data Availability

Data will be made available on request.
