# Supplementary material for: Ligand-dependent pharmacokinetic modulation via copper doping in ultrasmall gold nanoparticles
Source: Mater Today Bio. 2026 Mar 11;37:103009. doi: 10.1016/j.mtbio.2026.103009 (PMC13014963; doi:10.1016/j.mtbio.2026.103009)
Supplement: Multimedia component 1 [file mmc1.docx]

***Supplementary Material***

Ligand-Dependent Pharmacokinetic Modulation via Copper Doping in Ultrasmall Gold Nanoparticles

Jingqi Gong^1^, Jiali Tian^1^, Nengjie Wang, Di Huang*, Yuanli Liu* and Bing Tang*

Assoc. Prof. B. Tang, Dr. D. Huang

College of Chemistry and Bioengineering, Guilin University of Technology, Guilin 541006, People’s Republic of China

Prof. Y. Liu

College of Materials Science and Engineering, Guilin University of Technology, Guilin 541006, People’s Republic of China

E-mail addresses: [cbetb@glut.edu.cn](mailto:cbetb@glut.edu.cn) (B. Tang), [hd@glut.edu.cn](mailto:hd@glut.edu.cn) (D. Huang) and [lyuanli@glut.edu.cn](mailto:lyuanli@glut.edu.cn) (Y. Liu).

^1^ These authors contributed equally.

**Experimental Section**

**Calculation of Ligand Surface Density**

The ligand surface density was calculated based on thermogravimetric analysis (TGA) and transmission electron microscopy (TEM) data, assuming spherical metal cores.

Constants:

Avogadro constant: *N*_A_ = 6.022 × 10^23^ mol^−1^

Density of Au: *ρ*_Au_ =19.32 g cm^−3^

Density of Cu: *ρ*_Cu_ =8.96 g cm^−3^

Molecular weights:

*M*_GSH_ = 307.32 g mol^−1^

*M*_PEG−SH_ = 1000 g mol^−^**^1^**

**Alloy Density Calculation**:

For AuCu alloy nanoparticles, the density was calculated using the mass-fraction mixing rule:

$$\rho_{alloy}=\left( \frac{w_{Au}}{\rho_{Au}} + \frac{w_{Cu}}{\rho_{Cu}} \right)^{-1}$$

where: $w_{Au}$and $w_{Cu}$are the mass fractions of Au and Cu in the metal core, $\rho_{Au}$and $\rho_{Cu}$are the densities of pure Au and Cu.

Alloy density of GS-AuCuNPs: *ρ* = 18.00 g cm^−3^

Alloy density of PEG-AuCuNPs: *ρ* = 17.84 g cm^−3^

For a spherical nanoparticle:

$$V=\frac{4}{3}\pi r^{3}$$

$$A=4\pi r^{2}$$

Number of nanoparticles:

$$N_{NP}=\frac{m_{metal}}{\rho V}$$

Number of ligands:

$$N_{lig}=\frac{m_{lig}}{M_{lig}}N_{A}$$

Ligands per nanoparticle:

$$N_{lig/NP}=\frac{N_{lig}}{N_{NP}}$$

Surface density:

$$\sigma=\frac{N_{lig/NP}}{A}$$

**Representative Calculation (GS–AuNPs)**

TGA: ligand fraction = 29.5%; metal residue = 67.0%; sample mass = 4.752 mg

Metal mass:

$$m_{metal}=4.752\times0.67=3.184\text{ }\mathrm{mg}$$

Ligand mass:

$$m_{lig}=4.752\times0.295=1.402\text{ }\mathrm{mg}$$

TEM: diameter = 1.9 nm, $r=0.95\text{ }\mathrm{nm}$

Core volume:

$$V=\frac{4}{3}\pi(0.95\times{10}^{-7})^{3}=3.591\times{10}^{-21}\mathrm{cm}^{3}$$

Mass of one NP:

$$m_{NP}=19.32\times V=6.94\times{10}^{-20}\text{ }g$$

Number of nanoparticles:

$$N_{NP}=\frac{3.184\times{10}^{-3}}{6.94\times{10}^{-20}}=4.59\times{10}^{16}$$

Number of GSH molecules:

$$N_{GSH}=\frac{1.426\times{10}^{-3}}{307.32}\times N_{A}=2.75\times{10}^{18}$$

Ligands per NP:

$$N_{GSH/NP}=59.9$$

Surface area:

*A* = 11.341 nm^2^

Surface density:

*σ* = 5.28 molecules nm^−2^

The ligand surface densities of the remaining samples were calculated using the same method based on their respective TGA metal residues, molecular weights, densities, and TEM-derived diameters.

**Supplemental Tables and Figures**

Table S1. Parameters used for calculating ligand surface density of the nanoparticles.

| Sample | Mass (mg) | Metal Residue (%) | Core Diameter (nm) | Ligands/NP | Surface Density (molecules·nm^-2^) |
| --- | --- | --- | --- | --- | --- |
| GS–AuNPs | 4.752 | 67.0 | 1.9 | 59.9 | 5.28 |
| GS–AuCuNPs | 5.011 | 64.0 | 1.9 | 60.6 | 5.34 |
| PEG–AuNPs | 4.974 | 28.5 | 1.8 | 86.6 | 8.52 |
| PEG–AuCuNPs | 5.137 | 23.8 | 1.8 | 102.0 | 10.02 |


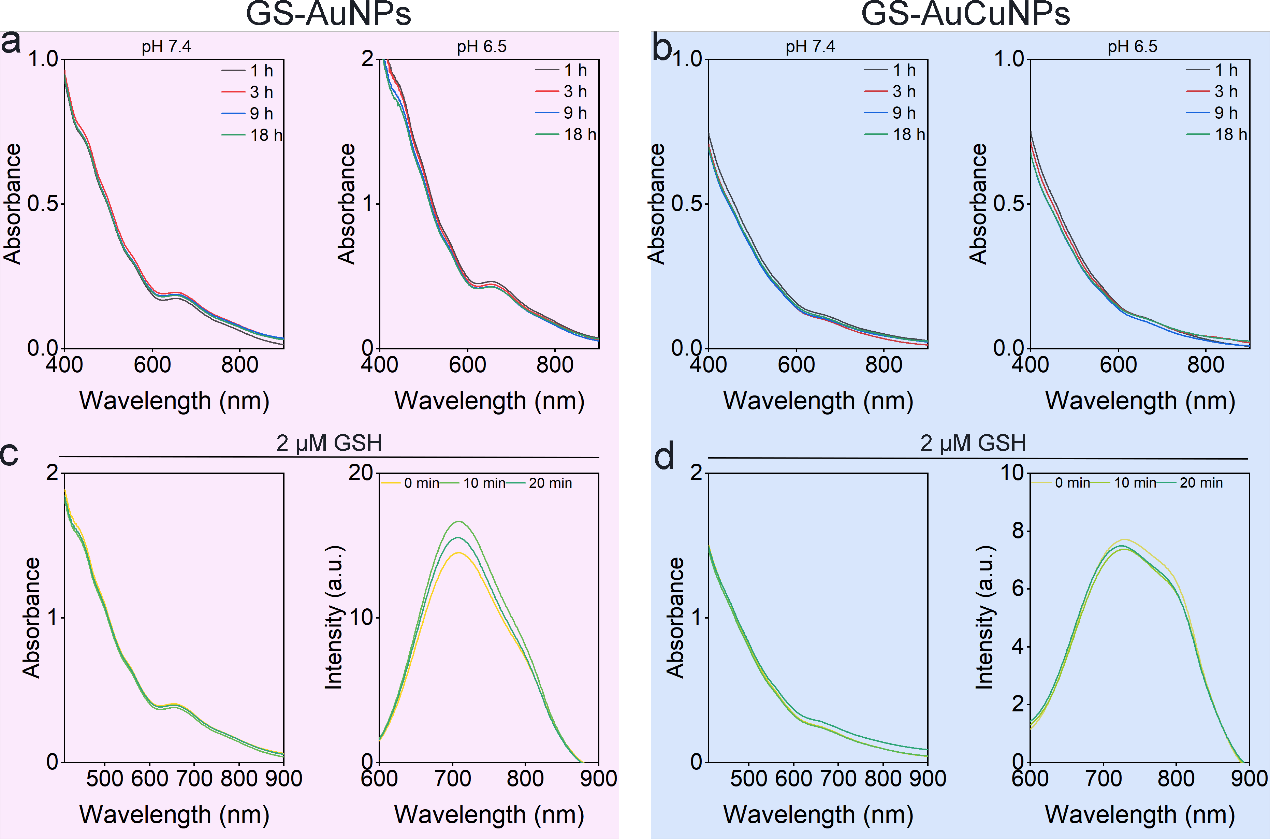


Fig. S1. Stability of GS-AuNPs and GS-AuCuNPs under different conditions. (a, b) UV-vis absorption spectra of (a) GS-AuNPs and (b) GS-AuCuNPs incubated at pH 7.4 and pH 6.5 for 1, 3, 9, and 18 h. (c, d) Absorption and emission spectra of (c) GS-AuNPs and (d) GS-AuCuNPs in the presence of 2 μM GSH over time. Both GS-AuNPs and GS-AuCuNPs remained colloidally stable at pH 7.4 and pH 6.5 for up to 18 h, with no significant changes in their UV–vis spectra. In addition, incubation with blood-relevant GSH concentrations (2 μM) induced no obvious spectral variation. These results confirm that GSH surface modification effectively maintains nanoparticle stability under physiological, mildly acidic, and reductive conditions.


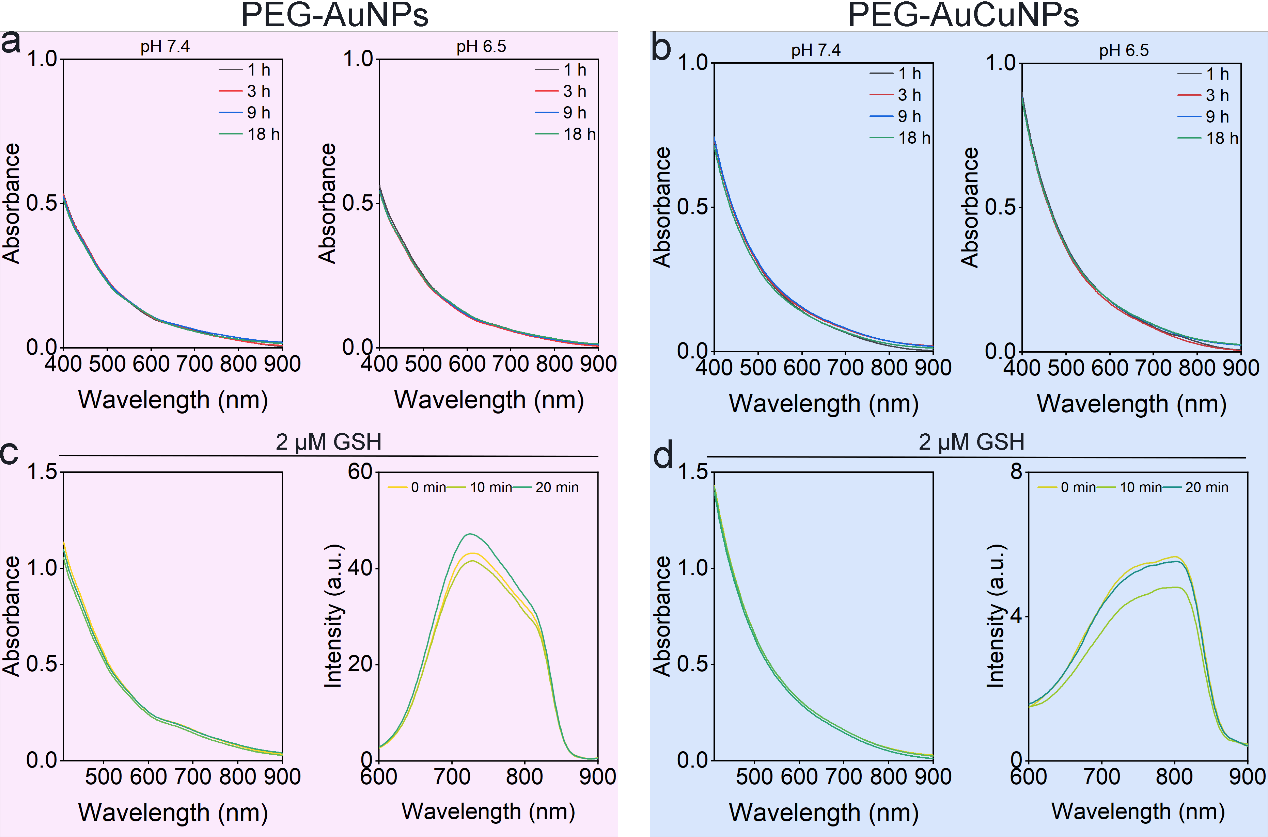


Fig. S2. Stability of PEG-AuNPs and PEG-AuCuNPs under different conditions. (a, b) UV-vis absorption spectra of (a) PEG-AuNPs and (b) PEG-AuCuNPs incubated at pH 7.4 and pH 6.5 for 1, 3, 9, and 18 h. (c, d) Absorption and emission spectra of (c) PEG-AuNPs and (d) PEG-AuCuNPs in the presence of 2 μM GSH over time. Both PEG-AuNPs and PEG-AuCuNPs remained colloidally stable at pH 7.4 and pH 6.5 for up to 18 h, with no significant changes in their UV–vis spectra. In addition, incubation with blood-relevant GSH concentrations (2 μM) induced no obvious spectral variation. These results confirm that PEG surface modification effectively maintains nanoparticle stability under physiological, mildly acidic, and reductive conditions.


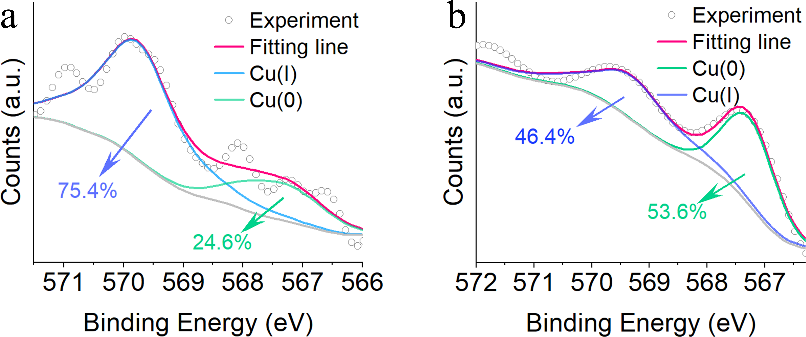


Fig. S3. Cu LMM Auger spectra of (a) GS-AuCuNPs and (b) PEG-AuCuNPs. Quantitative analysis showed that Cu(I) accounted for 75.4% in GS-AuCuNPs compared to 46.4% in PEG-AuCuNPs, suggesting ligand-dependent modulation of copper oxidation states.


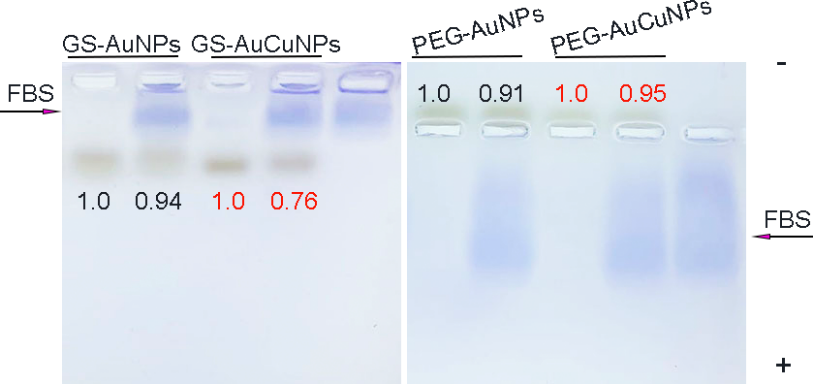


Fig. S4. Gel electrophoresis analysis of nanoparticle interactions with serum proteins. GS-AuNPs and GS-AuCuNPs were incubated with (+) or without (−) fetal bovine serum (FBS). The grayscale intensity at the original migration position relative to that before serum incubation was quantified. GS-AuCuNPs exhibited lower intensity retention than GS-AuNPs, indicating that enhanced COO^–^–metal coordination and increased -NH₂ exposure from copper doping modulate interfacial properties and alter serum protein interactions. PEG-coated nanoparticles show comparable retention regardless of copper doping. These results demonstrate ligand-dependent modulation of serum protein interactions upon copper incorporation.

Table S2. Pharmacokinetic parameters of ultrasmall nanoparticles obtained from two-compartment model fitting.

| **Parameters (unit)** | **GS-AuNPs** | **GS-AuCuNPs** | **GS-AuNPs with Cu^2+^** | **PEG-AuNPs** | **PEG-AuCuNPs** |
| --- | --- | --- | --- | --- | --- |
| Initial concentration  (C0, % ID/g) | 23.6±3.3 | 25.9 ±  1.1 | 24.4 ±  3.8 | 48.8 ±  5.6 | 55.4 ±  4.7 |
| Distribution half-life  (t1/2α, min) | 3.4 ±  0.35 | 4.2 ±  0.27 | 2.2 ± 0.21 | 12.6 ± 3.6 | 12.8 ± 2.1 |
| Elimination half-life  (t1/2β, h) | 3.8 ±  0.56 | 5.7 ±  0.43 | 2.1 ±  0.02 | 2.34 ±  0.27 | 2.63 ±  0.09 |
| Area under the curve  (AUC, % ID·h/g) | 127.8 ±  10.3 | 212.9 ±  8.9 | 73.9 ±  10.7 | 164.5 ±  28.5 | 210.1 ±  11.7 |
| Plasma clearance  (*CL*, mL/h) | 0.79 ±  0.06 | 0.47 ±  0.02 | 1.37 ±  0.21 | 0.62 ±  0.1 | 0.48 ±  0.03 |
| Volume of distribution  (*V*_d_, mL) | 4.3 ±  0.64 | 3.9 ±  0.17 | 4.2 ±  0.69 | 2.1 ±  0.25 | 1.8 ±  0.15 |
| Tumor accumulation  (% ID/g) | 1.65 ±  0.07 (1 h)/  0.93 ±  0.08(12 h) | 2.65 ±  0.24(1 h)/  1.40 ±  0.07(12 h) | NA | NA | NA |


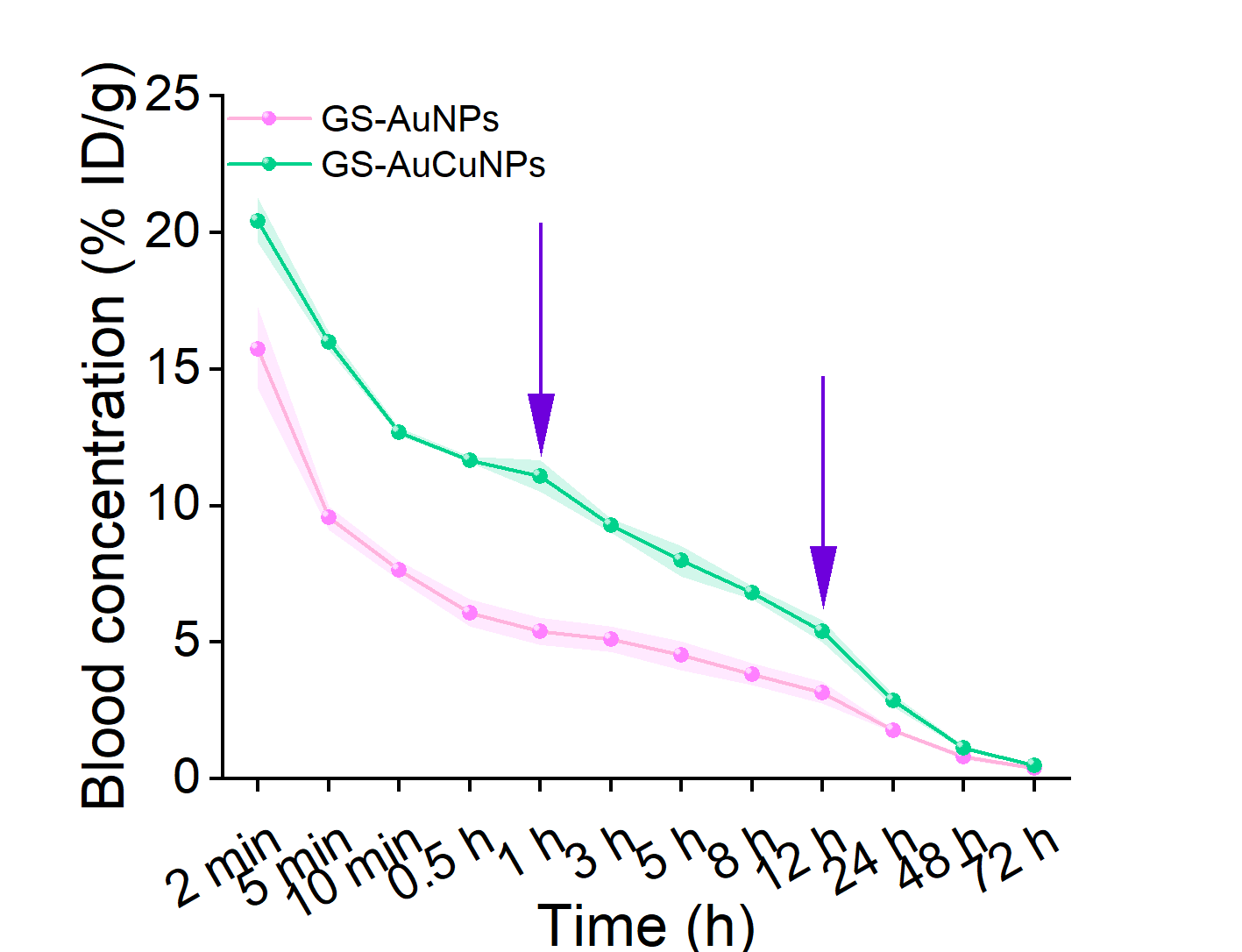


Fig. S5. Blood circulation profiles (%ID/g) over time for GS-AuNPs, GS-AuCuNPs.


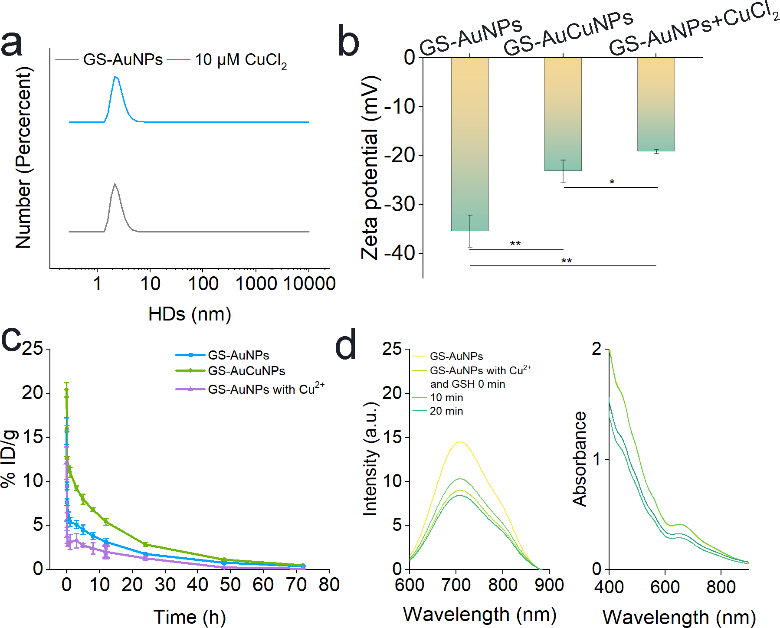


Fig. S6. Control experiments evaluating the effect of free Cu^2+^ on GS-AuNPs. (a) Hydrodynamic diameter (HD) distributions of GS-AuNPs before and after incubation with 10 μM CuCl_2_. (b) Zeta potential of GS-AuNPs, GS-AuCuNPs, and GS-AuNPs with CuCl_2_ (GS-AuNPs + Cu^2+^). (c) Pharmacokinetic profiles for GS-AuNPs, GS-AuCuNPs, and GS-AuNPs + Cu^2+^. (d) Emission and absorption spectra of GS-AuNPs + Cu^2+^ incubation with GSH (2 μM). Data are presented as mean ± SD (n = 3). Two-tailed unpaired Student’s t-test. ns, not significant; **p* < 0.05; ***p* < 0.01. Control experiments were performed to distinguish between the effects of alloying and simple Cu^2+^ coordination. Dynamic light scattering showed no change in the HD of GS-AuNPs + CuCl_2_ and GS-AuNPs. Zeta potential shifted toward a less negative value after CuCl_2_ incubation, yet this treatment did not improve blood exposure, indicating that a surface potential shift alone is insufficient to reproduce the pharmacokinetic benefit of alloyed Cu incorporation. GS-AuNPs + Cu^2+^ exhibited a significant decrease in AUC (73.9 ± 10.7 %ID·h·g⁻¹), showing reduced systemic exposure compared to GS-AuCuNPs under identical injection conditions. Unlike the stability of GS-AuCuNPs in blood GSH, GS-AuNPs + Cu^2+^ displayed unstable absorption and fluorescence spectra, suggesting that copper-induced pharmacokinetic enhancement in GS-AuCuNPs requires alloy formation during synthesis, not post-synthesis copper chelation.


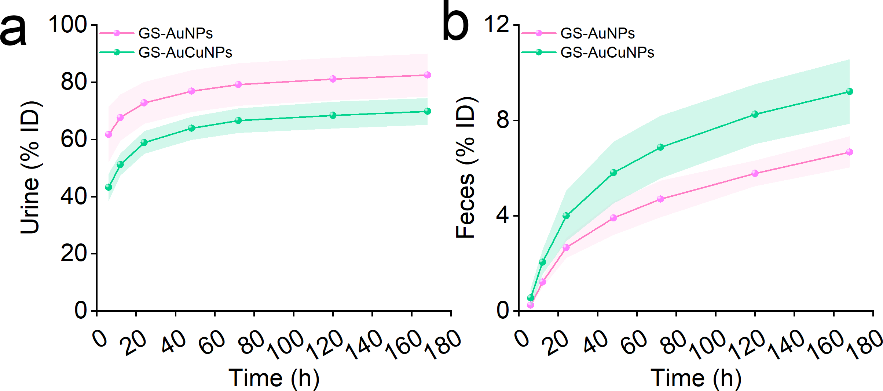


Fig. S7. Cumulative urinary and fecal excretion profiles over 168 h post-injection.


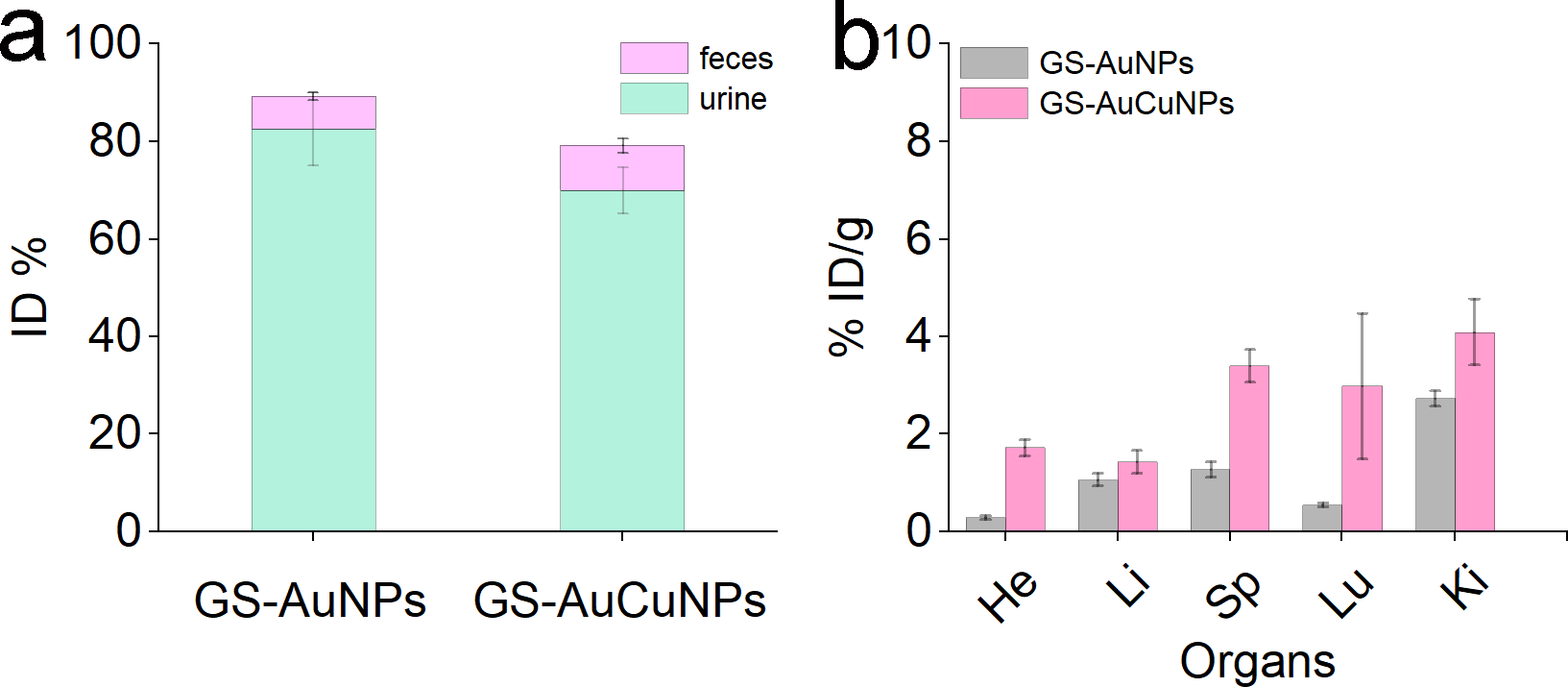


Fig. S8. Metabolism (a) and biodistributions (b) of the GS-AuCuNPs and GS-AuNPs at 168 h post-injection. The high clearance and low organ enrichment collectively indicate favorable biosafety profiles of the nanoparticles.


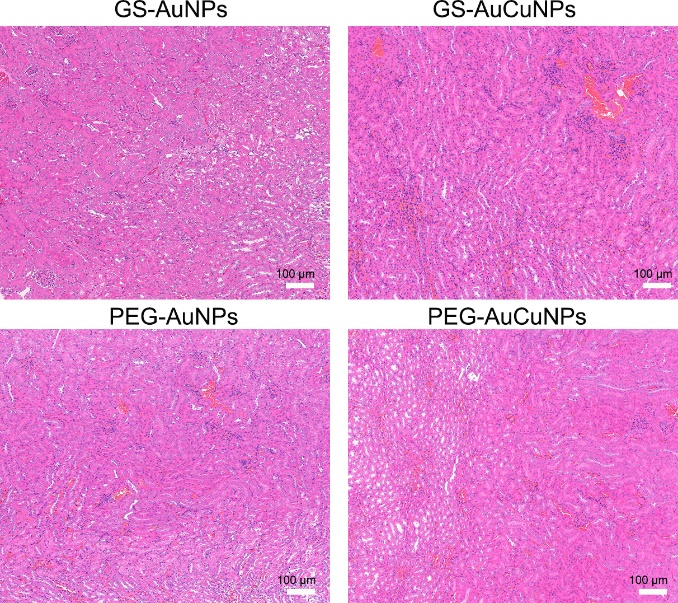


Fig. S9. Histological hematoxylin and eosin (H&E) staining images of the kidney after i.v. injection of the nanoparticles at 24 h p.i. The kidney exhibited complete histological morphology and no obvious lesions, indicating that GSH-coated and PEGylated nanoparticles showed negligible toxicity.


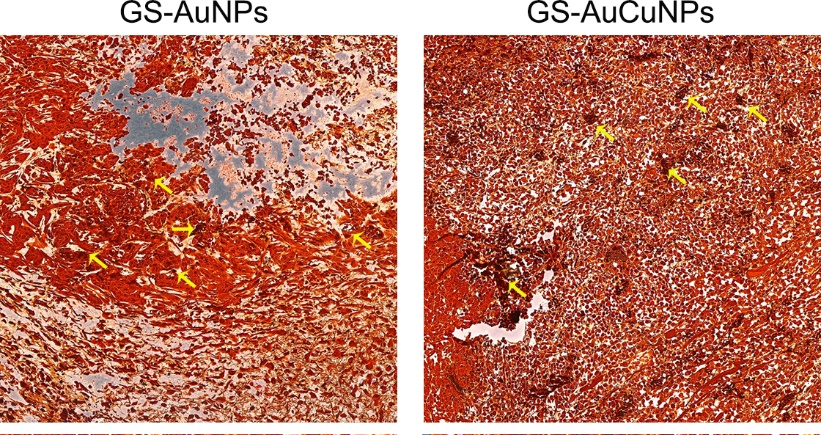


Fig. S10. Silver staining of the tumor tissues after i.v. injection of GS-AuNPs and GS-AuCuNPs at 24 h p.i. Yellow arrows indicate the position of the silver-stained nanoparticles.


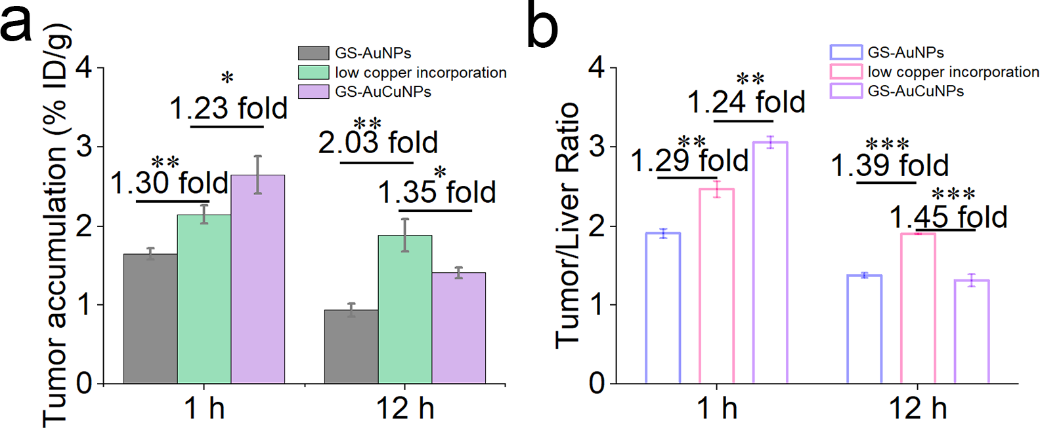


Fig. S11. Tumor accumulation (a) and tumor-to-liver ratios (b) of GS-AuNPs and GSH-coated AuCu nanoparticles with low and high Cu incorporation (GS-AuCuNPs) at 1 h and 12 h p.i. At 1 h p.i., both low Cu incorporation and GS-AuCuNPs exhibited higher tumor accumulation than GS-AuNPs, with the GS-AuCuNPs showing the greatest enhancement. Notably, the lower-Cu incorporation nanoparticle was synthesized using a reduced Cu precursor input (from 39 to 13 μL, 20 mM) relative to the GS-AuCuNP preparation. By 12 h p.i., although overall tumor accumulation decreased compared with 1 h, both Cu-doped formulations remained higher than GS-AuNPs, and the low-Cu group exhibited relatively greater retention among the alloyed samples. Consistently, tumor-to-liver ratios for all Cu-doped nanoparticles exceeded those of GS-AuNPs at 1 h and remained above 1 at 12 h p.i., indicating preferential tumor retention over hepatic accumulation. Collectively, these results demonstrate that copper incorporation reproducibly enhances tumor accumulation and targeting specificity with GSH modification, and that this effect can be tuned by adjusting copper content, supporting a composition-sensitive, ligand-dependent targeting behavior rather than an incidental outcome.
